# Persistent corneal blood staining after microhook trabeculotomy: A case report

**DOI:** 10.1097/MD.0000000000029278

**Published:** 2022-07-08

**Authors:** Ryota Aoki, Shunsuke Nakakura

**Affiliations:** a Department of Ophthalmology, Saneikai Tsukazaki Hospital, Himeji, Japan.

**Keywords:** case report, corneal bloodstaining, hyphema, microhook trabeculotomy, MIGS

## Abstract

**Introduction:**

Hyphema, that is, massive anterior chamber hemorrhage, is one of the major complications after a recent minimally invasive glaucoma surgery. Hyphema along with high intraocular pressure increases the risk of corneal bloodstaining.

**Patient Concerns:**

A 71-year-old female was receiving 0.1% fluorometholone drops in both eyes for severe dry eye. She was also receiving antiplatelet agents for platelet aggregation hyperactivity and prednisolone for interstitial pneumonia internally. Her right eye was suffering from increased intraocular pressure.

**Diagnosis:**

We diagnosed her right eye as steroid-induced glaucoma.

**Interventions:**

We performed microhook trabeculotomy.

**Outcomes:**

At postoperative day 10, she had total anterior chamber hemorrhage and high intraocular pressure, and subsequently developed corneal blood staining at postoperative day 15, for which we performed anterior chamber cleaning. After that, we did not perform additional anterior chamber cleaning, and during the 1-year follow-up, a gradual improvement was noted in the entire cornea with reddish-brown opacity, from the periphery to the center. However, almost the entire pupil was still covered with opacity, and her visual acuity was at the light perception at the final visit.

**Lessons:**

Corneal bloodstaining takes a considerable time to resolve and causes severe vision loss. Special attention should be given to persistent corneal blood staining when hyphema and high intraocular pressure are observed after minimally invasive glaucoma surgeries.

## 1. Introduction

Corneal bloodstaining is a complication that can occur when intraocular pressure (IOP) increases under the conditions of prolonged massive anterior chamber hemorrhage and can occur after trauma or internal eye surgery. An IOP of >25 mm Hg for 5 days may cause corneal blood staining.^[1]^ Corneal blood staining is clinically characterized by reddish-brown or gray opacity attributed to hemoglobin or its breakdown products.^[2]^ The opacity usually improves from the periphery by phagocytosis, but it takes a few years for the cornea to become completely clear, and in some cases, the corneal opacity may never resolve.^[3]^ Recently, minimally invasive glaucoma surgery (MIGS) has become popular globally, and a major complication of MIGS is hyphema. Most of the hyphema resolves over a period of several weeks and does not harm corneal transparency.

Herein, we report a case of persistent corneal blood staining after microhook ab interno trabeculotomy (μLOT)^[4]^ and raise caution for MIGS.

## 2. Case report

**Figure 1.**
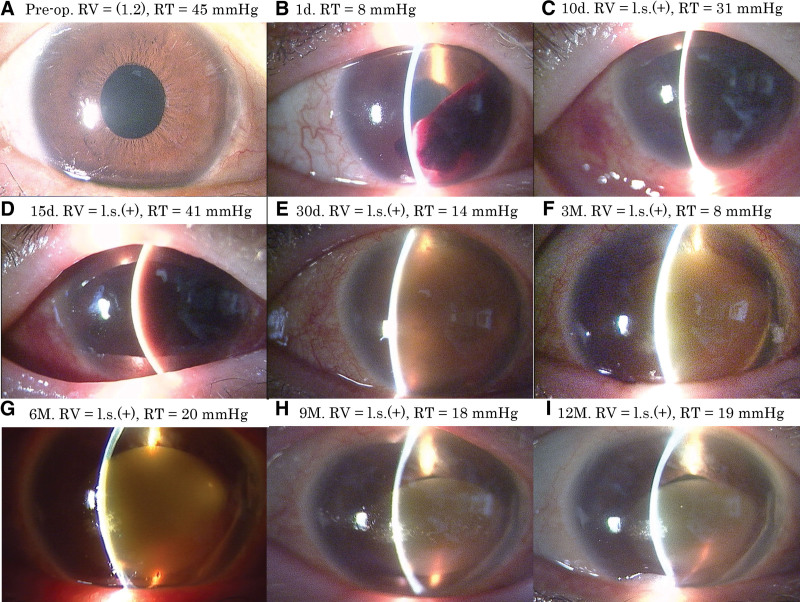
Images of slit-lamp photography pre- and postsurgeries. (A) Preoperative image. (B) Image obtained at 1 day after μLOT. A clot can be seen in the anterior chamber on the inferior nasal side. (C) Image obtained on day 10. Hyphema increased to the whole anterior chamber. (D) Image obtained on day 15. No improvement in hyphema was seen, and the cornea showed reddish-brown opacity. Anterior chamber irrigation was performed. (E) Image obtained on day 30. Corneal transparency was observed only in the entire circumferential corneal limbus. (F–H) Anterior segment findings at postoperative 3, 6, and 9 mo, respectively. The opacity tended to improve from the corneal limbus to the center. However, visual acuity was light perception. (I) Image obtained at postoperative month 12 (final visit). The pupil area was still slightly observable in the 12 o’clock direction. μLOT = microhook ab interno trabeculotomy, l.s.= light sense, RT = right ocular tension, RV = right vision.

A 71-year-old female was referred to our hospital for high IOP. She was receiving 0.1% fluorometholone drops four times daily in both eyes for severe dry eye associated with Sjogren’s syndrome. She was also receiving antiplatelet agents for platelet aggregation hyperactivity and prednisolone for interstitial pneumonia internally. She had already been treated with four components of glaucoma eye drops for steroid-induced glaucoma in her right eye, for which her best corrected visual acuity was 1.2 and IOP was 45 mm Hg. Her right cornea was clear with no inflammatory cells in the anterior chamber and without any pseudoexfoliation material deposition at the pupillary margin (Figure 1A). The anterior chamber angle was Grade 0 per the Scheie classification, and no peripheral anterior synechiae was observed. To reduce IOP, we conducted μLOT in the nasal and inferior angle using a microhook inserted into the Schlemm’s canal to incise the inner wall of the Schlemm’s canal and trabecular meshwork for 6 clock hours. Toward the end of the surgery, the anterior chamber was cleaned. No intraoperative complications were observed. The IOP on postoperative day 1 was 11 mm Hg, and a clot was observed in the anterior chamber (Figure 1B). Subsequently, 1.5% levofloxacin and 0.1% fluorometholone eye drops were started at 4 times daily. On postoperative day 8, the anterior chamber hemorrhage worsened, and the IOP increased to 57 mm Hg. Therefore, we additionally initiated oral acetazolamide at 500 mg/d. On postoperative day 10, the IOP decreased to 31 mm Hg, but anterior chamber hemorrhage remained unchanged compared with that at day 8 (Figure 1C). Because sufficient IOP reduction was not achieved, acetazolamide dose was increased to 750 mg/d, and brimonidine 0.1% eye drops were additionally initiated. However, on postoperative day 15, the IOP increased to 41 mm Hg, and corneal blood staining was observed (Figure 1D). On the same day, anterior chamber irrigation was performed, and the remaining hematoma in the anterior chamber was removed using a 23-gage anterior vitreous cutter. The next day, the IOP was 15 mm Hg, but corneal blood staining was observed throughout the cornea. Subsequently, 1.5% levofloxacin and 0.1% betamethasone eye drops were administered 4 times daily, and acetazolamide medication was continued at 750 mg/d. On postoperative day 23 (8 d after anterior chamber irrigation), the IOP was 11 mm Hg, and thus, acetazolamide was discontinued. On postoperative day 30, only the peripheral corneal ring became transparent (Figure 1E). After 3 months postsurgery, the opacity decreased gradually toward the central cornea (Figure 1F, 1G, 1H). At postoperative month 12 (final visit), the pupil area was slightly visible in the 12 o’clock direction (Figure 1I); however, the corneal opacity was still observed, and visual acuity was still light perception. During the follow-up, the patient was treated with levofloxacin 4 times daily, 0.1% betamethasone 4 times daily, 0.05% latanoprost, and 0.1% brimonidine eye drops in the right eye. Betamethasone was administered 4 times until 6 months postoperatively and thrice thereafter, and glaucoma eye drops were continued until 12 months after μLOT. IOP in the right eye remained between 9 and 19 mm Hg.

## 3. Discussion

Hyphema is a common complication of MIGS resulting from the incision of the trabecular meshwork. Tanito et al^[4]^ reported that of the 560 eyes that underwent μLOT alone and simultaneous cataract surgery, 30% had anterior chamber hemorrhage with niveau (hyphema), and 5% required anterior chamber washout. In the study by Mori et al,^[5]^ hyphema was observed in 16% of patients who underwent a single μLOT surgery, and the hyphema resolved without anterior chamber washout in all eyes. In addition, hyphema was observed in 31.8% of patients who underwent additional standard cataract surgery.^[6]^

Corneal blood staining is a serious complication as it can take as long as 2–3 years for the cornea to become clear, and in some cases, corneal opacity may remain not resolve completely.^[3]^ Although the criterion that IOP of ≥25 mm Hg for 5 days may cause corneal blood staining has been established,^[1]^ 3 cases that did not meet this criterion have been reported.^[7,8]^ Of these, 2 cases had corneal blood staining without elevated IOP after blunt trauma,^[7]^ and the third case was of corneal blood staining after cataract surgery with intraoperative posterior capsule rupture and iris hemorrhage.^[8]^

Reportedly, a decrease in endothelial function is a risk factor for blood penetration into the corneal stroma, which increases the risk of corneal blood staining.^[2,8]^

In the present case, there was no increase in IOP on postoperative day 1; however, on postoperative day 8, the IOP increased to 57 mm Hg along with increased hemorrhage in the anterior chamber. At this point, the patient may have met the criteria of IOP of ≥25 mm Hg for 5 days. We also suspected that if IOP was extremely high, corneal blood staining may have occurred in short time because intraocular barrier was already collapsed. As previously reported, corneal blood staining improved from the corneal limbus to the center of the cornea.^[8]^ However, 12 months after the surgery, the pupil area was only slightly transparent in the 12 o’clock direction, and visual acuity was not improved. In this case, it would have been appropriate to perform anterior chamber irrigation on postoperative day 8. In addition, the possibility of increased or prolonged anterior bleeding should have been considered since the patient was receiving antiplatelet medication.

If hyphema is observed after internal eye surgery or trauma, great care must be taken to prevent subsequent development of corneal blood staining, especially in the presence of high IOP. Early anterior chamber irrigation should be considered if the amount of hyphema is expected to be high or in the case of patients with impaired endothelial function.

## 4. Conclusion

We report a case of persistent corneal blood staining after μLOT. Our findings reveal that attention should be paid for anterior chamber hemorrhage with high IOP after MIGS.

## Author Contributions

Technical assistance for this manuscript was provided by R.A. Data collection was provided by R.A. Writing of this manuscript was provided by R.A. Editing assistance for this manuscript was provided by S.N.
